# Emerging Roles of USP18: From Biology to Pathophysiology

**DOI:** 10.3390/ijms21186825

**Published:** 2020-09-17

**Authors:** Ji An Kang, Young Joo Jeon

**Affiliations:** 1 Department of Biochemistry, Chungnam National University College of Medicine, Daejeon 35015, Korea; wldksdl555@naver.com; 2 Department of Medical Science, Chungnam National University College of Medicine, Daejeon 35015, Korea

**Keywords:** USP18, ubiquitin, ISG15, deubiquitinases, post-translational modifications, interferon signaling

## Abstract

Eukaryotic proteomes are enormously sophisticated through versatile post-translational modifications (PTMs) of proteins. A large variety of code generated via PTMs of proteins by ubiquitin (ubiquitination) and ubiquitin-like proteins (Ubls), such as interferon (IFN)-stimulated gene 15 (ISG15), small ubiquitin-related modifier (SUMO) and neural precursor cell expressed, developmentally downregulated 8 (NEDD8), not only provides distinct signals but also orchestrates a plethora of biological processes, thereby underscoring the necessity for sophisticated and fine-tuned mechanisms of code regulation. Deubiquitinases (DUBs) play a pivotal role in the disassembly of the complex code and removal of the signal. Ubiquitin-specific protease 18 (USP18), originally referred to as UBP43, is a major DUB that reverses the PTM of target proteins by ISG15 (ISGylation). Intriguingly, USP18 is a multifaceted protein that not only removes ISG15 or ubiquitin from conjugated proteins in a deconjugating activity-dependent manner but also acts as a negative modulator of type I IFN signaling, irrespective of its catalytic activity. The function of USP18 has become gradually clear, but not yet been completely addressed. In this review, we summarize recent advances in our understanding of the multifaceted roles of USP18. We also highlight new insights into how USP18 is implicated not only in physiology but also in pathogenesis of various human diseases, involving infectious diseases, neurological disorders, and cancers. Eventually, we integrate a discussion of the potential of therapeutic interventions for targeting USP18 for disease treatment.

## 1. Introduction

Ubiquitin conjugation to substrates (ubiquitination) is the most powerful and tightly fine-tuned post-translational modification (PTM) of proteins. Ubiquitination plays essential roles in regulating activity, interaction, subcellular localization, intracellular trafficking, and stability of proteins, thereby orchestrating a plethora of biological processes, involving stress responses, immune modulation, signaling transduction, control of cell cycle, division, and proliferation [1]. Ubiquitin possesses eight ubiquitination sites, seven internal lysine residues and a primary amine at the N-terminus, all of which participate in versatile polyubiquitin chain formation and result in distinct chain topologies [2,3]. Eight homotypic polyubiquitin chains can be generated via the extension on lysine residues or the N-terminal methionine residue of monoubiquitin conjugated to substrate. Additionally, heterotypic polyubiquitin chains can be generated by the formation of mixed ubiquitin chains or branched ubiquitin chains, in which ubiquitin can be modified on multiple sites [3,4,5,6,7,8]. Moreover, new chain types are emerging. In addition to chemical PTMs of ubiquitin, such as phosphorylation and acetylation [9,10,11], several ubiquitin-like proteins (Ubls) such as interferon (IFN)-stimulated gene 15 (ISG15) [12,13,14,15], small ubiquitin-related modifier (SUMO) [16] and neural precursor cell expressed, developmentally downregulated 8 (NEDD8) [17] [18,19] have been demonstrated to modify polyubiquitin chains [18,20,21,22], all of which could change the characteristics of ubiquitin and affect not only ubiquitin interactions but also formation and length of polyubiquitin chain, thereby generating sophisticated and versatile ubiquitin code.

Deubiquitinases (DUBs) disassemble the complex ubiquitin code and switch off the ubiquitin signal, which deals with the matched complexity of ubiquitination. In humans, more than one hundred DUBs have been identified. DUBs are composed of six structurally distinct families of different cysteine and metalloproteases. Five families of cysteine proteases include the ubiquitin C-terminal hydrolases (UCHs) with four members, the ovarian tumor proteases (OTUs) with 16 members, the Josephin family with four members, the motif interacting with ubiquitin (MIU)-containing novel DUB family (MINDYs) with four members, and the ubiquitin-specific proteases (USPs) with 54 members [1,23,24]. The sixth family of DUBs is Zn-dependent JAB1/MPN/MOV34 metalloproteases (JAMMs) with 16 members [23].

ISG15 contains two Ubl domains that have a sequence homology with ubiquitin. Both the N- and C-terminal Ubl domains of ISG15 show a remarkable similarity in their tertiary structures to ubiquitin, although the primary sequences of the N- and C-terminal Ubl domains share only 29 and 31% identities with ubiquitin, respectively [25]. The PTM of target proteins by ISG15 (ISGylation) is achieved through a sequential enzymatic cascade that is catalyzed by E1 activating enzyme UBE1L (UBA7), E2 conjugating enzyme UbcH8 (UBE2L6), and E3 ligases [13,15]. In a different way to how some Ubls are constitutively expressed, ISG15 is strongly upregulated by IFNs, retinoic acid, lipopolysaccharide (LPS), bacterial and viral infection or genotoxic stress-inducing agents [13,26,27,28,29,30], indicating that ISGylation is a tightly fine-tuned process. In addition, ISGylation can be reversed by specific isopeptidases. Ubiquitin-specific protease 18 (USP18), originally referred to as UBP43, with a calculated molecular weight of 43 kDa that was first discovered during the analysis of differentially expressed genes in acute myeloid leukemia 1 (AML1)-eight twenty-one (ETO) knock-in mice [31] and later in the analysis of virus-infected porcine alveolar macrophages [32] and human melanoma cell lines [33], is the major DUB that reverses ISGylation [34,35,36]. Additionally, *Usp18* has been characterized in various species and demonstrated to possess interferon-sensitive response elements (ISREs) in its promoter region and to be strongly induced by type I IFNs [32,33,37]. Of note, it has been demonstrated that USP18 is a multifaceted protein that not only reverses ubiquitination or ISGylation and disassembles the code generated by ubiquitination or ISGylation as a deconjugating enzyme but also plays a pivotal role in the negative regulation of type I IFN signaling, irrespective of its catalytic activity. Accordingly, USP18 has been suggested to be involved in versatile cellular processes, including signal transduction pathways, stress responses and responses to viral and bacterial infections [38,39,40,41,42,43,44].

The role of USP18 is obviously more complex than initially thought. In the present review, we discuss not only the basic biology and properties of USP18 but also the multifaceted roles of USP18. We also highlight new insights into how USP18 is implicated not only in physiology but also in pathophysiology of various human diseases involving infectious diseases, neurological disorders and cancers, suggesting the potential of therapeutic interventions for targeting USP18 for disease treatment.

## 2. Characteristics of USP18

### 2.1. Properties of USP18

**Figure 1:**
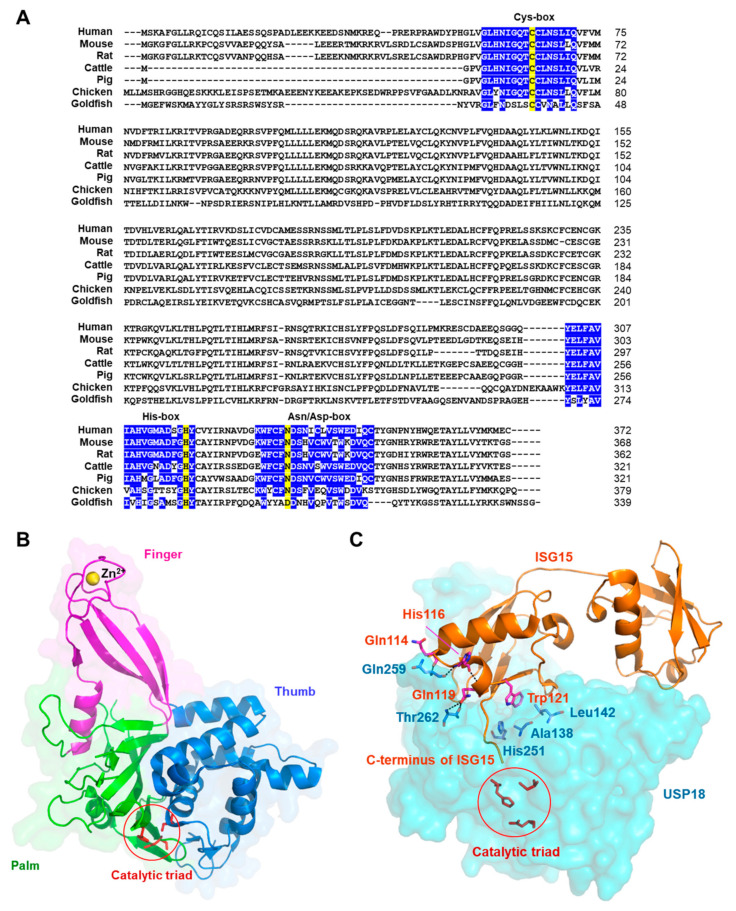
Characteristics of ubiquitin-specific protease 18 (USP18). (**A**) Comparison of amino acid sequence and sequence alignments of conserved domains of USP18 from various species. A catalytic triad composed of a cysteine, a histidine and asparagine/aspartate residues is shown in yellow. Conserved catalytic cores of USP18 from various species are displayed in blue. Cys-box contains the catalytic cysteine residue, His-box contains the catalytic histidine residue and Asn/Asp-box contains the catalytic asparagine/aspartate residue. (**B**) Structure of USP18 in the interferon-stimulated gene 15 (ISG15)-unbound state showing the three-dimensional architecture with finger, palm and thumb domains (PDB 5CHT). The catalytic triad is shown in red. A zinc ion bound to the finger domain is shown as sphere. (**C**) Structure of USP18 in the ISG15-bound state (PDB 5CHV). USP18 interacts with the C-terminal ubiquitin-like protein (Ubl) domain of ISG15 and the C-terminal tail of ISG15 lies in a cleft between the palm and thumb domains. Alanine at position 138 (Ala138), leucine at position 142 (Leu142) and histidine at position 251 (His251) residues within USP18 are involved in hydrophobic interactions with ISG15. Threonine at position 262 (Thr262) and glutamine at position 259 (Gln259) residues within USP18 form hydrogen bonds with glutamine at position 114 (Gln114), histidine at position 116 (His116) and glutamine at position 119 (Gln119) residues within ISG15.

*Usp18* was originally termed *Ubp43* since it encodes a protein with a calculated molecular weight of 43 kDa, homologous with ubiquitin processing proteases (UBPs). USP18 was first discovered during the analysis of differentially expressed genes in AML1-ETO knock-in mice [31] and later in virus-infected porcine alveolar macrophages [32] and human melanoma cell lines [33]. As a gene encoding a major deISGylating enzyme [34,35,36], *Usp18* has been characterized in various species and demonstrated to possess ISREs in its promoter region and therefore be strongly induced by IFNs [32,33,37]. USP18 possesses cysteine and histidine boxes, essential for enzymatic activity, which are highly conserved in USPs [31,34,39,45] (Figure 1A).

Human *Usp18* maps to chromosome 22q11.2. In humans, two isoforms of USP18 were discovered [46]. Compared to full-length USP18, N-terminal truncated form of USP18 is generated through translation from a non-canonical rare start codon CUG and is evenly distributed in both the cytoplasm and nucleus. The truncated form of USP18 functions as a main nuclear deISGylating enzyme. Interestingly, both of the two isoforms play roles not only dependent of enzymatic activity but also independent of deconjugating activity.

It has been demonstrated that homozygous *Usp18* knockout mice with additional breeding within a C56/B6 and 129 mixed background show a nearly normal lifespan, while homozygous *Usp18* knockout mice in an initial C57/B6 and 129 mixed strain show a decreased lifespan due to neurological abnormalities related to brain ependymal cell death resulting in aqueduct stenosis and hydrocephalus [47]. Additionally, homozygous *Usp18* knockout mice, obtained from backcrossing heterozygous *Usp18* knockout mice into a C57/B6 background, die before E15.5. However, *Usp18* knockout mice within the FVB/N background show a normal lifespan, indicating that the differences in the phenotype of *Usp18* knockout mice might result from the genetic background of these mice [42].

### 2.2. Structural Analysis of USP18

The crystal structures of mouse USP18 alone and in complex with mouse ISG15 have been solved. Similar to other cysteine proteases, the catalytic domain of USP18 possesses a common three-dimensional architecture that is characterized by finger, palm and thumb domains [48] (Figure 1B). The finger domain of USP18 interacts with a zinc ion. Additionally, USP18 harbors a catalytic triad composed of a cysteine, a histidine and an asparagine residue that is located at the interface between thumb and palm domain. Interestingly, the catalytic triad of USP18 is kept in an inactive conformation in the absence of ISG15 binding. Of note, given that USP18 is a multifaceted protein that plays a role not only in an isopeptidase-dependent but also in an isopeptisase-independent manner, it is intriguing that unlike other USPs, USP18 does not possess protein interaction domains [43].

It has been shown that USP18 recognizes the C-terminal Ubl domain of ISG15 [48]. All three domains of USP18, finger, palm and thumb domains are involved in the accommodation of C-terminal Ubl domain of ISG15 and the C-terminal tail of ISG15 is located in a cleft between palm and thumb domains, resulting in its access to catalytic triad (Figure 1C). Intriguingly, the specificity of USP18 for ISG15 is accomplished by the hydrophobic interactions between a hydrophobic patch in USP18 and a distinct hydrophobic region in ISG15 that is centered around tryptophan residue at position 121 and is not present in ubiquitin [43,48]. Additionally, the hydrophobic surfaces on USP18 and ISG15 are evolutionarily conserved between different mammals. Alanine at position 138, leucine at position 142 and histidine at position 251 residues within USP18 generate a hydrophobic pocket and with the aid of glutamine at position 139 and serine at position 192 residues, the three residues are involved in the formation of hydrophobic interactions with the hydrophobic patch of ISG15, suggesting that the preferential recognition and high specificity of USP18 toward ISG15 could be achieved by the hydrophobic interactions between USP18 and ISG15. Moreover, threonine at position 262 and glutamine at position 259 residues within USP18 form hydrogen bonds with glutamine at position 114, histidine at position 116 and glutamine at position 119 residues within ISG15, resulting in higher affinity of USP18 toward ISG15 [39].

### 2.3. Regulation of USP18 Expression

Expression of USP18 has been detected at different levels in several tissues. The upregulation of USP18 has been demonstrated in the thymus, spleen and liver, whereas its lower but clearly detectable expression has been described in adipose tissue, bone marrow and lung [31,35]. Additionally, higher expression of USP18 was detected in CD169+ macrophages and bone marrow-derived dendritic cells [49,50].

*Usp18* is strongly upregulated by type I and type III IFNs [33,35,37,51,52], lipopolysaccharides [53,54], polyI:C [53], tumor necrosis factor α (TNFα) [54], or genotoxic stresses [35,55]. Additionally, *Usp18* is remarkably induced after viral or bacterial infection [52,56,57,58].

Interestingly, USP18 has been shown to be degraded through the ubiquitin–proteasome system (UPS) [59]. SKP (S-phase kinase-associated protein)-Cullin-F-box protein (SCF^SKP2^) facilitates USP18 ubiquitination and subsequent degradation by the proteasomes. Moreover, ISG15-deficient patients show downregulation of USP18 and subsequently higher and persistent IFN signature [60]. Mechanistically, the association of USP18 with free intracellular ISG15 prevents SCF^SKP2^-mediated USP18 ubiquitination and subsequently its proteasomal degradation, thereby leading to the prevention of autoinflammation and over-amplification of IFN, which suggests that the long-term stabilization of USP18 by free intracellular ISG15 is essential for the negative feedback regulation of IFN signaling [60]. On the contrary, murine USP18 has been shown not to be dependent on ISG15 for its stabilization [61].

## 3. Multifaced Functions of USP18

### 3.1. USP18 as a deISGylating Enzyme

**Figure 2:**
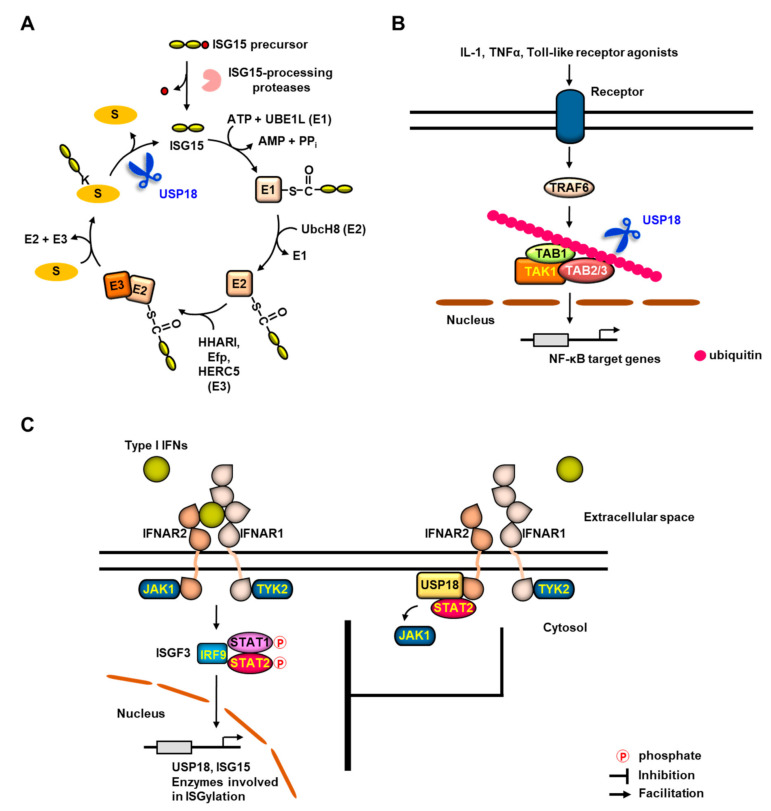
Multiple functions of USP18. (**A**) USP18 as a deISGylating enzyme. The expressions of USP18, ISG15 and enzymes involved in a three enzymatic cascade for the PTM of target proteins by ISG15 (ISGylation) are strongly induced by type I and type III interferons (IFNs), viral and bacterial infection, and genotoxic stresses. ISG15 exists as an immature precursor with a molecular weight of 17 kDa and is proteolytically processed into its mature form with a molecular weight of 15 kDa, resulting in the exposure of a carboxyl-terminal leucine-arginine-leucine-arginine-glycine-glycine (LRLRGG) motif that is required for ISGylation of target proteins. In a similar manner to ubiquitination, ISGylation utilizes a three-step enzymatic reaction. ISG15 is activated by E1 activating enzyme UBE1L at the expense of ATP and is subsequently bound to UBE1L via thioester bond. Following activation, ISG15 is transferred to the active-site cysteine of the E2 conjugating enzyme UbcH8 and then to a target protein with the aid of E3 ligase, such as human homolog of Ariadne (HHARI), estrogen-responsive finger protein (Efp) and E3 ubiquitin protein ligase 5 (HERC5). A major deISGylating enzyme, USP18 disassembles the specific code generated by ISGylation. USP18 functions in the reversal of ISGylation by cleaving off ISG15 that is conjugated to the target protein via isopeptide bond. (**B**) Negative regulation of nuclear factor κB (NF-κB) signaling by USP18. USP18 deubiquitinates transforming growth factor β (TGFβ)–activated kinase 1 (TAK1)/TAK binding protein 1 (TAB1) complex (TAK1/TAB1) complex and subsequently inhibits NF-κB signaling. (**C**) Regulation of type I IFN signaling by USP18. Type I IFN-stimulated dimerization of interferon α/β receptor 1 (IFNAR1) and interferon α/β receptor 2 (IFNAR2) facilitates interactions between Janus family kinases, JAK1 and tyrosine kinase 2 (TYK2) and IFNARs, thereby resulting in the activation of kinase activity of JAK1 and TYK2 through cross-phosphorylation, which provides docking sites for other effector proteins, signal transducer and activator of transcription proteins (STATs). Interferon-regulatory factor 9 (IRF9) associates with phosphorylated STAT1 and STAT2 and forms a transcription factor, interferon-stimulated gene factor 3 (ISGF3) complex, which recognizes and binds to interferon-sensitive response elements (ISREs) on the promoter regions of *Isgs*, thereby resulting in the induced expression of USP18, ISG15 and enzymes involved in ISGylation. Of note, type I IFN-induced USP18 plays a negative feedback regulator of type I IFN signaling. USP18 reduces the cell surface-binding affinity of type I IFNs. Further, STAT2-facilitated recruitment of USP18 to IFNAR2 competes with and displaces JAK1 from IFNAR2, which attenuates type I IFN signaling, subsequently leading to the inhibition of downstream expression of ISGs.

ISG15, the product of IFN-stimulated gene 15, is the first discovered Ubl [12,13,14,15,62,63]. In a similar manner to ubiquitination, ISGylation is accomplished through a three enzymatic cascade of activities that is catalyzed by E1 activating enzyme UBE1L (UBA7) [26], E2 conjugating enzyme UbcH8 (UBE2L6) [64,65], and E3 ligases, involving human homolog of Ariadne (HHARI) [66,67], estrogen-responsive finger protein (Efp; TRIM25) [68,69], or homologous to E6-AP C terminus (HECT) and regulator of chromosome condensation 1 (RCC1)-like domain (RLD) domain containing E3 ubiquitin protein ligase 5 (HERC5) [70,71] (Figure 2A). USP18 is the major DUB that reverses ISGylation [35,48]. Analysis of the specificity of USP18 using ^125^I-labelled ubiquitin and Ubls, involving ISG15, SUMO and NEDD8 has indicated that USP18 preferentially removes ISG15 from its conjugated target proteins [35]. Additionally, USP18 has been demonstrated not to show cross-reactivity toward ubiquitin [36,72], although it is becoming evident that USP18 can also recognize ubiquitin and remove ubiquitin from its conjugated proteins in a specific context. Accordingly, compared to wild-type mice, USP18-deficient mice show highly constitutive as well as upregulated IFN-inducible ISGylation without any changes in ubiquitination [47].

Studies using mass spectrometric analysis have led to the identification of several hundreds of candidate proteins that can be ISGylated [73,74]. However, only a subset of these candidate proteins has been validated to be ISGylated, characterized and functionally determined. Given that the functional consequences of protein ISGylation are closely associated with the regulation of various cellular processes, involving protein translation, autophagy, exosome secretion, DNA repair and immune modulation, it is pivotal to re-evaluate USP18 and to determine sophisticated and fine-tuned mechanisms in which USP18 is involved.

### 3.2. USP18 as a Deubiquitinating Enzyme

On the contrary to the original findings, USP18 has been demonstrated to remove ubiquitin from ubiquitinated proteins. It has been reported that USP18 interacts with transforming growth factor β (TGFβ)–activated kinase 1 (TAK1)/TAK binding protein 1 (TAB1) complex and deubiquitinates TAK1/TAB1 complex, thereby leading to the negative regulation of nuclear factor κB (NF–κB) signaling in T cells [75] (Figure 2B). Interestingly, USP18-deficient T cells have been demonstrated to be defective in T helper 17 (Th17) cells differentiation and to exhibit hyperactivation of NF-κB and nuclear factor of activated T-cells (NFAT), resulting in an increase in interleukin-2 (IL–2), which suggests the role of USP18 in T cell-mediated adaptive immune response and autoimmune disease. Moreover, USP18 attenuates the ubiquitination of the TAK1/TAB1 complex and IκB kinase α/β/NF–κB essential modulator (IKKα/β–NEMO) complex in an isopeptidase-dependent and -independent manner, respectively, resulting in the negative regulation of NF-κB signaling [55]. USP18 removes lysine 63-linked polyubiquitin chains from TAK1, while USP18 inhibits lysine 63-linked polyubiquitination of NEMO via direct binding to NEMO. Accordingly, USP18 depletion in human promonocytic THP–1 cells upregulates the expression of proinflammatory cytokines, involving tumor necrosis factor α (TNFα), IL–6 and IL–1β. Further, USP18 in hepatocytes associates with and deubiquitinates TAK1, which inhibits TAK1 activation and subsequently attenuates c-Jun N-terminal kinase (JNK) and NF-κB signaling, thereby leading to the amelioration of hepatic steatosis [76]. Interestingly, USP18 expression has been shown to be downregulated in the livers of nonalcoholic steatohepatitis (NASH) patients. Recently, it has been demonstrated that USP18 directly interacts with epithelial–mesenchymal transition (EMT)-inducing transcription factor, twist-related protein 1 (TWIST1) and removes ubiquitin from TWIST1 in its catalytic activity-dependent manner, thereby protecting TWIST1 from proteasome-mediated degradation [77].

### 3.3. Deconjugating Activity-Independent Role of USP18

Elevated and sustained type I IFN responses are detrimental, leading to an increase in inflammation [78,79,80]. As a result, multiple layers of feedback mechanisms to modulate IFN signaling could be required. It has been demonstrated that independent of its deconjugating activity USP18 negatively regulates type I IFN signaling [45] (Figure 2C). USP18 specifically associates with the second chain of type I IFN receptor subunit, IFN α/β receptor 2 (IFNAR2), and competes with Janus kinase 1 (JAK1) for binding to IFNAR2, thereby blocking the interaction between JAK and the IFN receptor and subsequently attenuating IFN signaling, which suggests the pivotal role of USP18 in the desensitization of type I IFN signaling after first wave of ISGs induction. Interestingly, the association of USP18 with IFNAR2 is not dependent on the dimerization of IFNAR2 with IFN α/β receptor 1 (IFNAR1) [81]. Moreover, it has been shown that USP18 interrupts JAK-mediated cytosolic interactions between IFNAR1 and IFNAR2 and shifts the equilibrium from IFNAR1–IFNAR2–IFNα ternary complex to IFNα–IFNAR2 binary complex, thereby leading to the downregulation of type I IFN signaling [51,81,82]. Intriguingly, it has been shown that USP18 is recruited to the IFNAR2 in a signal transducer and activator of transcription 2 (STAT2)-dependent manner and suppresses type I IFN signaling [83]. N- and C-terminal regions of USP18 are required for its association with STAT2, while a DNA-binding domain and a coiled-coil region of STAT2 are required for the interaction with USP18. Additionally, a homozygous missense mutation in *Stat2* found in an infant who died of autoinflammation is a gain of function (GOF) for induction of late IFN signaling [84]. Mechanistically, the mutant form of STAT2 has been demonstrated to fail to recruit USP18 to IFNAR2, thereby leading to the prevention of USP18-mediated downregulation of type I IFN signaling.

Given that USP18 plays multifaceted roles not only in the deISGylating activity-dependent manner but also in its deconjugating activity-independent manner, especially for the modulation of IFN signaling, the functions of USP18 should be interpreted with caution to discriminate deISGylation from the regulation of IFN signaling.

## 4. Implications of USP18 in Physiology and Pathophysiology

### 4.1. USP18 and Viral Infection

Elucidation for the role of USP18 in immune responses against viral infection is far more complicated, even though the importance of USP18 has become enormously pinpointed. It has been shown that USP18-deficient mice show IFN hypersensitivity and significant resistance to the cytopathic effects of vesicular stomatitis virus (VSV), lymphocytic choriomeningitis virus (LCMV) and sindbis virus (SINV), indicating the role of USP18 in innate immunity against viral infection [85]. Additionally, knock-in mice harboring enzymatically inactive USP18, USP18^C61A/C61A^ mice exhibited increased viral resistance against vaccinia virus and influenza B virus infections, while USP18^C61A/C61A^ mice did not exhibit IFN hypersensitivity, increased lethality or morphological abnormalities [86].

Host signaling pathways in modulating hepatitis virus infection remain to be elusive, while roles of viral proteins during infection are increasingly uncovered. Interestingly, USP18 has been demonstrated to play an important role in the host innate immune response to chronic viral infections such as hepatitis C virus (HCV), raising the possibility of the close association of USP18 with persistent HCV infection and non-responsiveness to IFN. IFNα-mediated upregulation of USP18 has been demonstrated to make the liver cells refractory to IFNα [87]. Furthermore, sustained expression of ISG15 has been shown to stabilize USP18 and negatively regulate IFN signaling, which facilitates pro-viral responses that are associated with chronic viral hepatitis [88,89], whereas depletion of USP18 has been demonstrated to potentiate anti-HCV activity of IFN by more than 40 fold [90], suggesting that at least in liver, upregulation of USP18 blunts type I IFN signaling and subsequently leads to an increase in HCV production. Furthermore, it has been demonstrated that chronic HCV patients with higher expression of USP18 failed treatment with standard pegylated IFNα-ribavirin [91,92,93]. Additionally, chronic hepatitis B patients with upregulated USP18 showed poor outcome after treatment of IFN [94]. Moreover, downregulation of USP18 has been reported not only to attenuate HBV replication but also to facilitate the clearance of HBV infection [95]. Intriguingly, USP18-deficient mice have been reported to be more viable and to have lower viral titers, compared to wild-type or UBE1L-deficient mice, indicating that the deISGylating activity-independent role of USP18 is involved in the anti-viral response against HBV infection. Recently, it has been demonstrated that USP18 upregulation in memory CD4 T cells (Mem) derived from human immunodeficiency virus 1 (HIV-1)-infected patients inhibits adequate cell survival and long-lasting maintenance in AKT, also known as protein kinase B (PKB)-dependent manner [96].

### 4.2. USP18 and Bacterial Infection

The role of USP18 in the control of bacterial infection has not yet been fully clarified. USP18-deficient mice showed hyperactive IFN signaling and subsequent less propagation of *Salmonella typhimurium* (*S. typhimurium*) [53]. Additionally, CD11c-Cre^+^ cells derived from USP18-deficient mice have been reported to mitigate bacterial titers in several organs and to prolong survival [97]. Interestingly, USP18 inhibition promotes the antibacterial effect of TNFα and subsequently induces reactive oxygen species (ROS), thereby controlling primary and secondary bacterial infection, which suggests the therapeutic potential of targeting USP18 in patients to ameliorate disease caused by serious bacterial infections. On the contrary, mice with a missense mutation in *Usp18*, *Usp18^Ity9^* showed the impairment of STAT4 phosphorylation and IFNγ production due to STAT1 hyperactivation, thereby leading to the exhibition of lethal susceptibility to *S. typhimurium* and higher bacterial titer in liver and spleen [98,99]. Of note, this phenotype seems to be due to the deficiency of USP18-mediated negative regulation of type I IFN signaling, rather than the deficiency of deISGylating activity of USP18, since both the USP18 and ISG15-deficient mice did not show improved survival after infection with *S. Typhimurium* [99].

### 4.3. USP18 and Cancer

Increasing evidence has begun to demonstrate that USP18 is associated with the pathogenesis of cancer, even though the role of USP18 in tumorigenesis is still controversial. Given that USP18 is involved in IFN signaling, USP18 could be linked to tumor progression. The expression of USP18 has been shown to be deregulated in lung cancer, breast cancer, bladder cancer and melanoma [38,40,100,101,102,103,104]. Recently, USP18 upregulation has been demonstrated in patients with hepatocellular carcinoma [105]. In mouse model of chronic myelogenous leukemia-like myeloproliferative disease, USP18 has been demonstrated to play an important role in the regulation of latency and severity of leukemia development [106]. Additionally, USP18 depletion in the breast cancer cell line resulted in an increase in chemotherapy and IFNα-induced apoptosis with robust activation of caspase-8 and caspase-3 [107]. USP18 depletion has also been shown not only to reduce acute promyelocytic leukemia cell growth but also to induce apoptosis [108]. Furthermore, it has been reported that upregulation of USP18 in glioblastoma cell lines is associated with the resistance to TNF-related apoptosis inducing ligand (TRAIL)-induced apoptosis after IFNα challenge [107,109]. Of note, USP18 has been demonstrated to be a positive regulator of epidermal growth factor receptor (EGFR) by inactivating microRNA-7 (miR–7) that is known to reduce the expression of *EGFR* mRNA in cancer cells [110]. miR-7 acts downstream of USP18 to regulate *EGFR* mRNA translation via the 3′-untranslated region (UTR). Accordingly, depletion of USP18 activates miR–7 and subsequently downregulates the expression of EGFR, thereby leading to the suppression of tumorigenesis and the facilitation of apoptosis of cancer cells.

The forced repression of USP18 is involved in the destabilization of cyclin D1 and promyelocytic leukemia/retinoic acid receptor α (PML–RARα), thereby leading to a reduction in proliferation and increased apoptosis [104,108,111]. Interestingly, the introduction of catalytically inactive mutant form of USP18 into USP18-depleted cancer cells did not show the same effect as the introduction of wild type USP18, indicating the importance of USP18 enzymatic activity in tumor progression [104]. USP18 has been shown to stabilize phosphatase and tensin homolog (PTEN) in both murine and human lung cancer cell lines [112]. Furthermore, USP18 deubiquitinates and stabilizes Kirsten rat sarcoma viral oncogene homolog (KRAS), thereby leading to the promotion in aggressiveness of lung cancer [101]. Of note, USP18 depletion leads to the mis-localization of KRAS from the plasma membrane and the destabilization of KRAS. Notably, loss of *Usp18* in *Kras^LA2/+^* mice has been reported to significantly attenuate lung cancer formation, compared to parental *Kras^LA2^*^/+^ mice harboring activated KRAS, pinpointing the implication of USP18 as a potential therapeutic target for cancer treatment. Additionally, USP18 depletion resulted in resistance to breakpoint cluster region protein (BCR)– Abelson (ABL)-meditated oncogenic transformation [106]. USP18-deficient bone marrow cells with BCR-ABL viral transduction/transplantation exhibited a significantly increased latency of disease development, whereas wild-type bone marrow cells with BCR-ABL viral transduction/transplantation exhibited the rapid development of a chronic myeloid leukemia (CML)-like myeloproliferative disease. Interestingly, this resistance to oncogenic transformation is largely due to the hypersensitivity of USP18-deficient cells to type I IFN signaling, raising the possibility that USP18 is pivotal for the control of the latency and severity of leukemia development and that abrogation of USP18-mediated downregulation of IFN signaling could potentiate the effects of type I IFNs against cancer development. Furthermore, USP18 has been demonstrated to activate oncogenic NF–κB signaling by disrupting ISGylation of NF–κB [113]. Recently, it has been shown that the expression of USP18 is upregulated in glioblastoma [77]. Interestingly, USP18 associates with TWIST1 and mediates the stabilization of TWIST1, thereby leading to glioblastoma cell migration and invasion. Moreover, higher USP18 expression is closely associated with poor prognosis in glioblastoma patients, indicating the critical role of USP18 in glioblastoma progression. Moreover, USP18 downregulation has been shown to be closely associated with a favorable prognosis of muscle invasive bladder cancer (MIBC) patients [102]. Cancer-specific survival in low USP18 expression group is longer than in high expression group, indicating that upregulated USP18 is significant risk factor for cancer-specific death.

The role of USP18 in tumor microenvironment has begun to be demonstrated. USP18 depletion has been demonstrated to result in the high production of T cell chemoattractant C-X-C motif chemokine ligand 10 (CXCL10), which recruits CD4^+^ T cells and leads to the creation of a tumor-suppressive microenvironment [114]. On the contrary, IFNγ-induced upregulation of USP18 in tumor cells plays a pivotal role not only in the inhibition of tumorigenesis but also in the maintenance of antitumor immunity and immunosurveillance [100]. Intriguingly, USP18 in B16 melanoma cells has been shown to attenuate tumor cell-mediated inhibition of T cell proliferation and suppress the expression of programmed cell death protein 1 (PD-1). Furthermore, USP18-deficient mice in an FVB/N background exhibited IFN-hypersensitive microenvironment and deregulated proliferation of vascular smooth muscle cells, thereby leading to the initiation of leiomyosarcoma [115].

To summarize, not only the development of selective modulators of USP18 but also the supply of the rationale for combination of USP18 modulation with conventional anticancer therapy might be worthy for the treatment of cancer.

### 4.4. USP18 and Neurological Disorders

It has been shown that dysregulation of type I IFN signaling in USP18-deficient mice is linked to neuropathological changes. Over-amplification of type I IFN signaling might be associated with the pathogenesis of various neurological disorders, even though type I IFN is helpful for the treatment of neurological diseases. USP18-deficient mice have been demonstrated to exhibit severe neurological phenotypes, involving tremor, loss of balance with early mortality, and convulsions [116]. Additionally, USP18-deficient mice exhibited necrosis of ependymal cells concurrent with hydrocephalus and reduced life expectancy [47]. Furthermore, constitutive activation of type I IFN signaling and subsequently elevated expression of various ISGs have been reported in USP18-deficient mice [117]. USP18-deficient mice have also been reported to possess clusters of microglia in white matter, which is common to microgliopathies. Intriguingly, these phenotypic alterations result from not the absence of deISGylating activity of USP18 but the prolonged activation of STAT1 and subsequently sustained type I IFN signaling independent of deISGylation, suggesting that USP18 deficiency leads to hypersensitized microglia with persistent activation of immune responses.

Multiple sclerosis (MS) is the most common autoimmune disease of the central nervous system. Genome-wide association studies have revealed that polymorphisms related to *Usp18* are linked to MS susceptibility and response to type I IFN [118,119,120]. Interestingly, one haplotype has been reported to be correlated with downregulation of USP18 and upregulation of clinical symptoms.

Pseudo-TORCH syndrome (PTS) resembles a congenital infection without any infectious agents and manifests with cerebral calcifications, enlarged ventricles and microcephaly. Clinical observations of two unrelated families with PTS demonstrated that patients harboring autosomal recessive loss-of-function mutations in *Usp18* exhibit neuropathological and neuroimmune phenotypes, involving persistent activation of STAT1 and remarkable induction of activated astrocytes and microglia, indicating the importance of USP18 in neuroimmune response and the involvement of USP18 in type I interferonopathy [121].

## 5. Concluding Remarks and Future Perspectives

**Figure 3:**
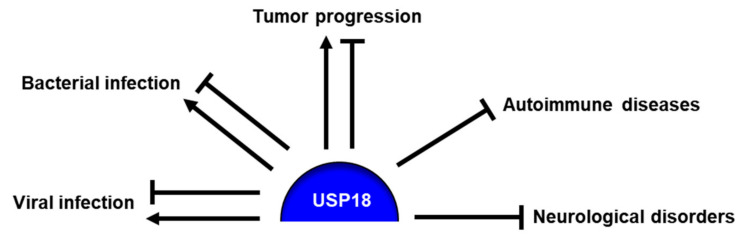
USP18 regulation of pathophysiology. USP18 is involved in the control of infectious diseases, autoimmune diseases, neurological disorders and cancers. USP18 attenuates the pathogenesis of autoimmune diseases and neurological disorders, whereas the role of USP18 in tumor progression and responses to viral and bacterial infections has not yet been completely addressed.

DUBs reverse versatile ubiquitin code generated by PTMs with matched complexity and precision of PTMs and switch off the signal. DUBs regulate various cellular processes and are associated with pathogenesis of diseases. Therefore, the improved understanding of DUBs will hopefully lead to the development of therapeutic strategies for the treatment of diseases. Among the six families of DUBs, USPs are the largest, with >50 members. New aspects of USP18 have been elucidated. However, the function of USP18 is obviously more sophisticated than initially thought. USP18 is a multifaceted protein that not only removes ubiquitin or ISG15 from conjugated proteins and disassembles the code as a deconjugating enzyme but also plays a pivotal role in the fine-tuning of type I IFN signaling irrespective of its catalytic activity. Of note, it has begun to be demonstrated that USP18 plays essential roles in the control of a large variety of cellular processes and subsequently in the development of pathogenesis, involving infectious diseases, interferonopathy, neurological disorders and cancers (Figure 3). Therefore, the identification of novel binding partners of USP18 that can modulate deconjugating activity-dependent or independent roles of USP18, the clarification of broad repertoire of physiological functions and the determination of molecular mechanisms in which USP18 is involved in the pathogenesis of diseases could be instrumental to providing a plethora of potential therapeutic strategies, thereby leading to the intervention of various human diseases.
